# Investigating the roles of loneliness and clinician- and self-rated depressive symptoms in predicting the subjective quality of life among people with psychosis

**DOI:** 10.1007/s00127-017-1470-1

**Published:** 2017-12-14

**Authors:** Piotr Świtaj, Paweł Grygiel, Anna Chrostek, Jacek Wciórka, Marta Anczewska

**Affiliations:** 1 First Department of Psychiatry, Institute of Psychiatry and Neurology, Sobieskiego 9, 02-957 Warsaw, Poland; 2 Institute of Pedagogy, Jagiellonian University, Cracow, Poland

**Keywords:** Psychosis, Quality of life, Loneliness, Depression

## Abstract

**Purpose:**

To examine the roles of loneliness and clinician- and self-rated depressive symptoms as predictors of the subjective quality of life (QoL) in psychosis.

**Methods:**

This cross-sectional study was conducted on a sample of 207 patients diagnosed with psychotic disorders. They were assessed with self-reported measures of QoL, loneliness and depression and with clinician-rated measures of depression and overall psychopathology. Multiple indicators multiple causes (MIMIC) modeling was used to analyze the data.

**Results:**

Both loneliness and depression turned out to be independent predictors of impaired QoL. However, once loneliness was accounted for, the effect of depression on QoL was markedly reduced and the effect of loneliness proved to be visibly larger. Self-rated depression was found to be more strongly associated with QoL than clinician-rated depression. Each type of depression measure explained a unique amount of variance in QoL. Depression moderated the relationship between loneliness and QoL in such a way that the negative effect of loneliness on QoL weakened with the increasing intensity of depressive symptoms.

**Conclusions:**

Therapeutic programs aiming to enhance the QoL of people with psychotic disorders should incorporate interventions targeting both loneliness and depression and need to be tailored to the clinical status of patients. The emphasis on alleviating loneliness should be placed first of all in the case of those with low levels of depression, among whom the negative impact of loneliness on QoL is especially strong. Researchers should be aware that the method chosen for assessing depressive symptoms in models predicting QoL in psychosis matters.

## Introduction

Loneliness is a subjective, negative experience resulting from a mismatch between the desired and actual quantity and/or quality of one’s social relationships. It is not synonymous with social isolation, which concerns the objective characteristics of the situation and refers to the absence of relationships with other people [1]. One can say that loneliness is perceived social isolation, rather than actual social isolation (for a comprehensive review of the conceptualizations of loneliness see, e.g., [2, 3]).

People with psychotic disorders have been consistently shown to experience more intense sense of loneliness than the general population or healthy control groups [4–7]. Existing studies have identified diverse clusters of factors that may contribute to this greater severity of loneliness in psychosis, including anhedonia and subjective thought disorder [5], positive symptoms [8], depression and poor self-reported social cognition [7], experiences of discrimination, diminished self-esteem and unwillingness to seek social support [9], internalized stigma, weak social support, restricted social network, poor interpersonal competence and a high number of psychiatric inpatient admissions [6], living arrangement (living in semi-independent and independent apartments as opposed to living in group homes) and low levels of social support and participation in the community [10], or low scores on measures of self-efficacy for community life, self-esteem, social network and community integration [11]. Despite these findings, there are also indications that people with psychotic disorders may find it difficult to report loneliness or to mention that this is a need to be met by mental health services, possibly because the experience of socialization is particularly stressful for them [12].

Loneliness is a highly clinically relevant issue since it has the potential to substantially impair an individual’s well-being and to increase the risk of numerous negative health outcomes, including substance abuse, sleep disturbances, cognitive decline, anxiety, depression, suicidality, poorer immune, endocrine and cardiovascular functioning, and increased mortality [2, 3, 13, 14]. Past research has already revealed that loneliness may negatively affect the quality of life (QoL) of people with schizophrenia and other serious mental illnesses [15–17]. The question remains unresolved, however, as to what extent the impact of the feelings of loneliness on the QoL of individuals diagnosed with psychotic disorders is independent of depression. On the one hand, depressive symptoms are among the dimensions of psychopathology most strongly influencing QoL in psychosis [18–20]. On the other hand, depression and loneliness are closely related and may share some common causes and features [2]. The specific nature of the relationship between loneliness and depression is still a matter of debate, since evidence on their prospective associations is so far mixed. For example, in a study by Caccioppo et al. [21] loneliness predicted subsequent changes in depressive symptomatology, but not vice versa, while the reverse was found by Lasgaard et al. [22]. There are also studies indicating that loneliness and depressive symptoms influence one another reciprocally [23, 24].

Because of the high correlations usually found between the two constructs, loneliness is sometimes even perceived as a symptom of depression and items referring to loneliness are included in several popular instruments measuring depression [24]. Thus, it is of theoretical interest and of practical importance to determine whether the negative effect of loneliness on QoL in patients with psychotic disorders would still hold after controlling for depressive symptoms, and if so, how the strength of the effects of loneliness and depression on QoL might compare to each other.

Furthermore, it should be noted that while loneliness is basically a subjective phenomenon, which is usually captured by self-report measures [1, 3], depression is commonly assessed using either self- or observer-rated instruments [25, 26]. It has been demonstrated that although self-ratings of depression can be a valid tool in the clinical assessment of patients with psychosis, in only about half of the patients do the self-ratings correspond well with the ratings of clinicians [27]. This raises other interesting questions. Will a clinician-rated and a self-rated measure of depressive symptoms explain a unique amount of variance in QoL? And which type of depression measure will be more strongly associated with QoL after accounting for loneliness? An analysis of these methodological issues may contribute to a better understanding of previous research findings regarding the relationships between depression, loneliness and QoL and may also inform future studies in this area.

Given the above, in this paper, our main purpose is to investigate the roles of loneliness and clinician- and self-rated depressive symptoms in predicting the subjective QoL in psychosis. Specifically, acknowledging that most research indicates that despite the considerable similarities between depression and loneliness they remain distinct phenomena [2], we hypothesize that higher levels of loneliness and depression will be independently related to worse QoL. As an exploratory part of the study, we compare the strength of the effect of loneliness with the effects of clinician- and self-rated depressive symptoms on QoL and test the possibility that measures of loneliness and depression interact in the prediction of QoL.

## Methods

### Participants

The study sample comprised people receiving psychiatric treatment in inpatient and day wards and an outpatient clinic of the Institute of Psychiatry and Neurology (IPN) in Warsaw (Poland) and who met the following inclusion criteria: (1) diagnosis of non-affective psychotic disorder (International Classification of Diseases, 10th Revision—ICD-10: F20–F29), (2) age over 18 years old, (3) written, informed consent to participate in the study, and (4) a stable mental condition, according to the treating psychiatrist, sufficient to enable the understanding and accurate answering of the questions in the questionnaires. Individuals with active drug or alcohol dependence, organic brain disease, severe cognitive deficits or documented intellectual disability (also known in some classification systems as mental retardation) were excluded. Patients were recruited between February 2013 and February 2015. Of the 267 persons asked to take part in the study, 207 (77.5%) agreed. The 60 patients who declined to participate did not differ significantly from the participants in terms of sex, age, type of psychiatric facility, or psychiatric diagnosis (all *P* > 0.05).

### Measures

#### Subjective quality of life

The Satisfaction With Life Scale (SWLS) [28] (Polish adaptation [29]) is a brief, five-item instrument measuring global life satisfaction. Items are scored by respondents on a scale from 1 (strongly disagree) to 7 (strongly agree). The greater the total score, the higher the level of satisfaction with life. In our sample, Cronbach’s *α* for the SWLS turned out to be 0.86.

#### Loneliness

The De Jong Gierveld Loneliness Scale (DJGLS) [30] (Polish validation [31]) consists of 11 items, to which interviewees are asked to respond using a five-point scale ranging from 1 (yes!) to 5 (no!). It can be used to assess both the overall level of loneliness and two of its dimensions: emotional (6 items) and social (5 items). After recoding the items referring to the emotional aspects of loneliness, a higher total score indicates a more intense global sense of loneliness. In this study, Cronbach’s *α* for the DJGLS was found to be 0.89.

#### Clinician-rated depression

The Calgary Depression Scale for Schizophrenia (CDSS) [32] (Polish adaptation [33]) is a structured interview scale composed of nine items, which is scored from 0 (absence of a symptom) to 3 (severe symptom). The time frame refers to the previous 2 weeks. A greater total score reflects more severe depression. The scale demonstrated good internal consistency (Cronbach’s *α* = 0.84).

#### Self-rated depression

The Center for Epidemiologic Studies Depression Scale-Revised (CESD-R) [34] contains 20 items referring to various symptoms of depression. Respondents are instructed to indicate how often they “have felt this way in the past week or so”. Response options range from 0 (not at all or less than 1 day) to 4 (nearly every day for 2 weeks). The higher the total score, the more severe the depressive symptoms. The Polish version of the scale was developed specifically for the purpose of this study using forward and back translations and a review by a bilingual panel of experts. The value of Cronbach’s *α* for the CESD-R was 0.93.

#### Psychopathological symptoms

The standard version of the Brief Psychiatric Rating Scale (BPRS) [35] (Polish version [36]) is one of the most popular tools measuring psychiatric symptoms. It includes 18 items rated by a clinician on a scale which ranges from 1 (symptom not present) to 7 (symptom extremely severe). It is the patients’ present condition that is assessed, on the basis of their verbal reports of symptoms experienced in the days preceding the interview and observation of their behavior during it. A higher total score implies a greater intensity of psychopathology. Cronbach’s *α* for the BPRS proved to be 0.91.

### Procedures

The ethical approval for the present study was granted by the Bioethical Committee of the IPN. In each participating setting, eligible patients were identified by staff psychiatrists. They were then approached by the members of the research team, who invited them to take part in the study. All participants provided their informed consent. Socio-demographic and clinical background data were collected on the basis of information obtained from respondents and analysis of available medical records. The self-administered questionnaires (SWLS, DJGLS and CESD-R) were completed by the patients in private. The observer-rated measures (CDSS and BPRS) were administered by a trained clinician.

### Statistical analyses

Preliminary analyses included calculating means (*M*) and standard deviations (SD) or percentages, as appropriate, for all study variables, Cronbach’s *α* coefficients for the instruments used, and Pearson product–moment correlations between the key constructs. Furthermore, independent samples *t* tests (for continuous variables) and *χ* ^2^ tests (for categorical variables) were carried out for comparison of background characteristics between the patients who took part in the study and those who refused to participate. As a main analysis, multiple indicators multiple causes (MIMIC) modeling [37, 38], which is a special case of structural equation modeling (SEM), was employed. It allows for the simultaneous testing of the factor structure and the effects of background variables. In this approach—unlike in ANOVA or MANOVA—the dependent variables are latent variables based on multiple indicators with appropriate control for measurement error [39]. The MIMIC model is composed of two parts: (1) a measurement component, which defines the relations between latent variables and their indicators, and (2) a structural component, which specifies the relationships among latent variables and covariates.

Initially, before the addition of covariates, confirmatory factor analysis (CFA) was carried out to confirm a one-factor structure of the measure of the subjective QoL (i.e., SWLS) found in previous studies [40]. CFA was performed using maximum likelihood estimation with robust standard errors (MLR). This estimation procedure is robust to violations of non-normality. To identify potential sources of misfit within a specified model, a more detailed evaluation of fit was obtained by inspecting the modification indices (MI) [41]. One of the main purposes of using the MI is the production of a better fit model [38]. This measure indicates how much *χ* ^2^ value will decrease if parameters (e.g., correlating measurement errors) are included.

Overall model fit was evaluated by means of the Yuan–Bentler (Y-B) scaled *χ* ^2^ statistic, root mean square error of approximation (RMSEA), comparative fit index (CFI), Tucker–Lewis index (TLI), Akaike’s information criterion (AIC), and the Bayesian information criterion (BIC). We assumed that a non-significant *χ* ^2^ test relative to the degrees of freedom (*df*) would indicate that the implied theoretical model significantly reproduces the sample variance–covariance relationships in the matrix. We also presumed that CFI and TLI values between 0.90 and 0.95 would represent the lower boundary of potentially acceptable fit [42] and that RMSEA values less than 0.08 would suggest adequate fit [43, 44]. The AIC and BIC were used to compare the fit of models with different amounts of parameters. These indices represent a trade-off between model accuracy and complexity. Lower values denote better fit [45].

**Fig. 1:**
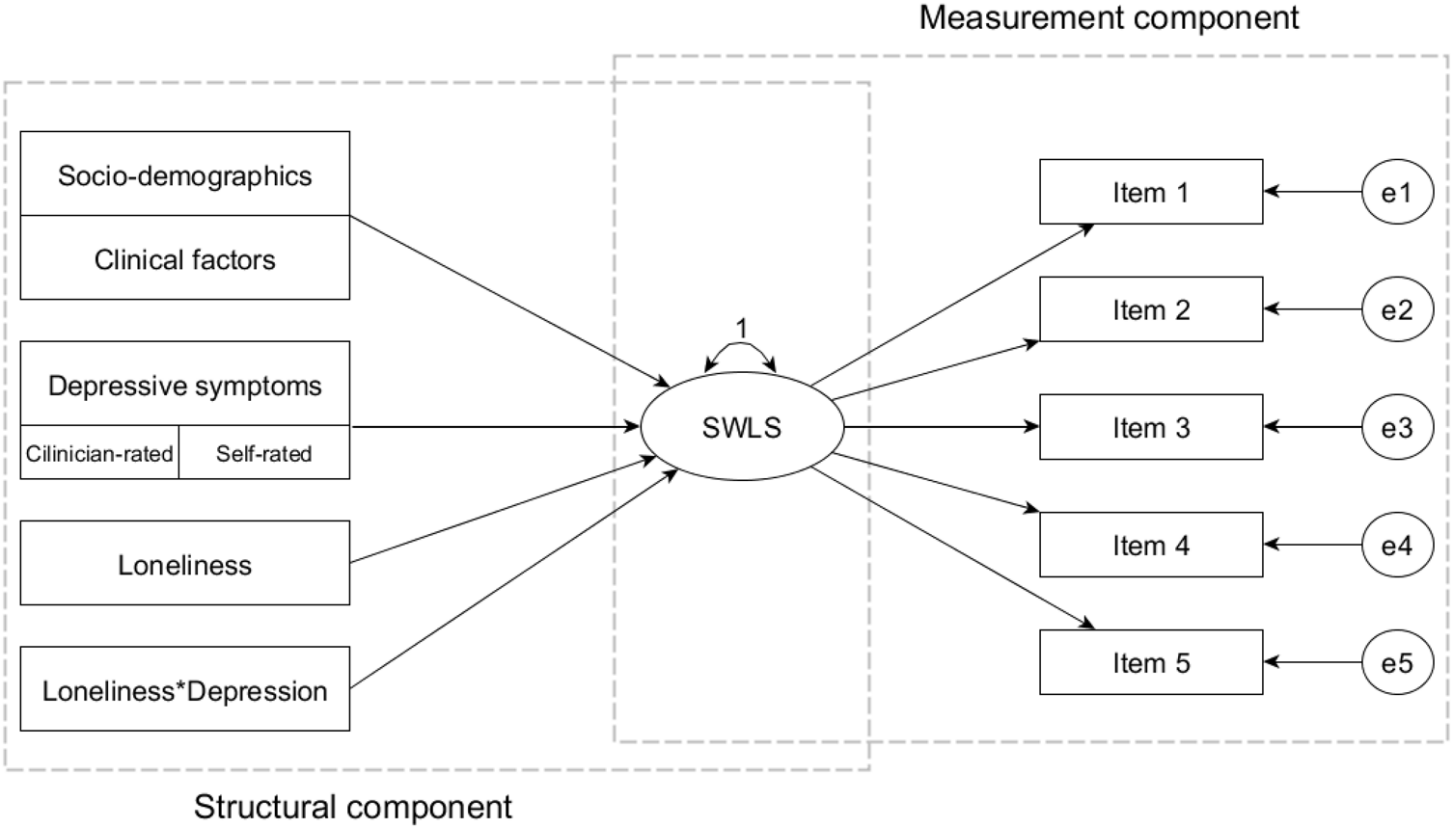
The multiple indicators multiple causes (MIMIC) model tested in the study.* SWLS* Satisfaction With Life Scale

Next, the effects of covariates on a hypothesized one-factor model of the SWLS were tested by means of the MIMIC modeling. The covariates were added to the model in the following order: (1) socio-demographic and clinical variables; (2) depressive symptoms (clinician- and self-rated); (3) loneliness; and (4) interaction effects between measures of loneliness and depression. Prior to analysis, measures of loneliness and depression were standardized to clarify the interpretation of interaction effects. The proposed MIMIC model is graphically illustrated in Fig. 1.

Missing values were rare. In the case of the SWLS, two participants failed to answer one item and one participant failed to answer two items. In the DJGLS, two respondents missed one item. As regards the CESD-R, four persons left one item unanswered, two persons missed two items, and one person did not respond to four items. No missing values were detected in the CDSS and BPRS. Little’s missing completely at random (MCAR) test [46] was conducted to check whether the missing data were MCAR (if any missing values were recorded). This supported the hypothesis that in the case of every analyzed scale data were MCAR: for the SWLS *χ* ^2^ = 4.39, *df* = 7, *P* = 0.73; for the DJLGS *χ* ^2^ = 30.29, *df* = 20, *P* = 0.07; and for the CESD-R *χ* ^2^ = 111.44, *df* = 90, *P* = 0.06.

To reduce the number of missing values, for all study instruments total scores were calculated by summing up the ratings of individual items and then dividing the total by the number of valid answers. Another advantage of this approach is that the overall score computed in such a way immediately provides an idea about the level of a measured construct. As far as the (latent) dependent variable is concerned, missing values were handled using full information maximum likelihood (FIML) estimation [47]. In FIML, the parameters (and standard errors) of a statistical model are estimated using a likelihood function for each participant based on observed relationships between non-missing data, without imputing or removing critical information from the data set [48].

The data were analyzed with the use of IBM SPSS Statistics 24.0 (SPSS Inc., Chicago, IL) and Mplus 7.4 [49]. *P* values of less than 0.05 were considered significant.

## Results

The sample had a mean age of 38.3 years (SD = 12.6) and was almost evenly split between men (50.2%) and women (49.2%). Most of the participants were single, never married (73.4%), unemployed (66.2%), had secondary or higher education (48.3 and 42.5%, respectively), and resided in a large city > 100,000 inhabitants (71.5%). Slightly above a quarter (27.5%) lived alone. The mean duration of illness was 12.9 years (SD = 11.0) and the mean number of psychiatric inpatient admissions was 6.0 (SD = 8.4). At the time of the research, 70% of the respondents were treated in inpatient wards and 30% were in outpatient or day care. The distribution of psychiatric diagnoses was as follows: schizophrenia, F20 (78.3%), persistent delusional disorder, F22 (3.4%), acute psychotic disorder, F23 (10.6%), schizoaffective disorder, F25 (6.8%), and unspecified nonorganic psychosis, F29 (1.0%).

Descriptive statistics and Pearson correlations for the instruments administered to study participants are presented in Table 1.

**Table 1:** Descriptive statistics and intercorrelations of the measures used in the study

Measure	*M* (SD)	Possible range	1	2	3	4
1. Satisfaction With Life Scale (SWLS)	3.35 (1.44)	1–7	–			
2. De Jong Gierveld Loneliness Scale (DJGLS)	2.83 (0.80)	1–5	− 0.52**	–		
3. Calgary Depression Scale for Schizophrenia (CDSS)	0.47 (0.44)	0–3	− 0.31**	0.41**	–	
4. Center for Epidemiologic Studies Depression Scale-Revised (CESD-R)	1.22 (0.91)	0–4	− 0.34**	0.47**	0.55**	–
5. Brief Psychiatric Rating Scale (BPRS)	2.13 (0.61)	1–7	− 0.08	0.13	0.21**	0.26**

For all instruments, higher scores indicate higher levels of the measured constructs

*M* mean, *SD* standard deviation

*P < 0.05, **P < 0.01

Scores on the instruments measuring QoL, loneliness, clinician-rated depression and self-rated depression were all significantly correlated in the expected direction. Global severity of psychopathology as assessed by the BPRS showed significant (positive) relationships only with both measures of depression. Importantly, the clinician- and self-rated depression scales turned out to be correlated at the level of *r* = 0.55, *P* < 0.01, which means that the CDSS and CESD-R shared slightly less than a third (30.3%) of the variance.

Table 2 displays the results of the CFA of the SWLS.

**Table 2:** CFA models of the Satisfaction With Life Scale (SWLS)

Items	Model 1			Model 2		
	Factor loadings	Intercepts	Residual variances	Factor loadings	Intercepts	Residual variances
1. In most ways my life is close to my ideal	1.40 (0.80)**	3.19	0.36	1.43 (0.82)**	3.19	0.34
2. The conditions of my life are excellent	1.17 (0.71)**	3.40	0.50	1.19 (0.72)**	3.40	0.48
3. I am satisfied with my life	1.51 (0.83)**	3.82	0.32	1.52 (0.83)**	3.82	0.31
4. So far I have gotten the important things I want in life	1.33 (0.74)**	3.34	0.46	1.26 (0.70)**	3.34	0.51
5. If I could live my life over, I would change almost nothing	1.25 (0.64)**	3.00	0.59	1.16 (0.59)**	3.00	0.65
Y-B *χ* ^2^ (*df*)		18.14 (5)**			5.72 (4)	
RMSEA (90% CI)		0.11 (0.06–0.17)			0.05 (0.00–0.12)	
CFI/TLI		0.95/0.91			0.99/0.99	
AIC/BIC		3722.60/3772.60			3709.91/3763.23	

Before parenthesis: unstandardized loadings; in parenthesis: standardized loadings

Model 2 allowed residual covariances between items 4 and 5. The correlation between these items was *r* = 0.40, *P* < 0.01

**P < 0.01

The initial single-factor CFA model (Model 1) did not fit the data well. Although high CFI (0.95) and TLI (0.91) values suggested acceptable model fit, the *χ* ^2^ was significant (*χ* ^2^ = 18.14, *df* = 5, *P* < 0.01) and the RMSEA exceeded 0.08 (RMSEA = 0.11, 90% CI 0.06–0.17), indicating the need for model modification. Inspection of the MI revealed that specifying item residual covariances between items 4 (So far I have gotten the important things I want in life) and 5 (If I could live my life over, I would change almost nothing) would considerably improve the fit of the model (MI = 13.30). Following the inclusion of this residual covariance (*r* = 0.40, *P* < 0.01), the modified model (Model 2) fitted the data satisfactorily. All goodness-of-fit statistics were adequate (*χ* ^2^ = 5.72, *df* = 4, *P* = 0.22; RMSEA = 0.05, 90% CI 0.00-0.12; CFI = 0.99; TLI = 0.99). In addition, lower values of the AIC and BIC statistics confirmed that Model 2 presented better data fit than Model 1. It should be noted that in both models all five items had salient (i.e., *λ* > 0.30) and statistically significant standardized factor loadings, meaning that the convergent validity of the single-factor structure was achieved. Based on this analysis, in the subsequent MIMIC models, we used a single-factor model with correlated error terms of items 4 and 5.

The effects of covariates on a hypothesized one-factor model of the SWLS are shown in Table 3.

**Table 3:** The effects of covariates on a hypothesized one-factor model of the Satisfaction With Life Scale (SWLS)

	Model 1	Model 2	Model 3	Model 4	Model 5	Model 6	Model 7	Model 8	Model 9	Model 10
**Socio-demographic and clinical variables**										
Gender^a^	0.05 (0.14)	− 0.06 (0.14)	− 0.06 (0.14)	− 0.09 (0.14)	− 0.04 (0.12)	− 0.04 (0.12)	− 0.06 (0.12)	− 0.04 (0.12)	− 0.05 (0.12)	− 0.07 (0.12)
Age	− 0.20 (0.10)	− 0.22 (0.11)*	− 0.16 (0.10)	− 0.18 (0.10)	− 0.21 (0.10)*	− 0.19 (0.09)*	− 0.19 (0.10)*	− 0.22 (0.10)*	− 0.18 (0.10)	− 0.19 (0.10)*
Education^b^										
Primary or vocational	− 0.51 (0.21)*	− 0.52 (0.19)**	− 0.34 (0.20)	− 0.38 (0.20)	− 0.27 (0.17)	− 0.20 (0.17)	− 0.22 (0.17)	− 0.28 (0.17)	− 0.27 (0.16)	− 0.29 (0.17)
Higher	0.16 (0.15)	0.14 (0.14)	0.13 (0.14)	0.13 (0.14)	0.08 (0.12)	0.08 (0.12)	0.08 (0.12)	0.06 (0.12)	0.02 (0.12)	0.02 (0.12)
Marital status^c^										
Single (never married)	− 0.77 (0.20)**	− 0.67 (0.18)**	− 0.67 (0.19)**	− 0.64 (0.18)**	− 0.66 (0.17)**	− 0.66 (0.17)**	− 0.64 (0.17)**	− 0.64 (0.16)**	− 0.67 (0.16)**	− 0.66 (0.16)**
Widowed/separated or divorced	0.06 (0.30)	0.17 (0.31)	0.07 (0.29)	0.13 (0.30)	0.12 (0.28)	0.07 (0.27)	0.10 (0.28)	0.14 (0.28)	0.07 (0.26)	0.09 (0.27)
Living circumstances^d^	− 0.17 (0.17)	− 0.27 (0.17)	− 0.22 (0.15)	− 0.26 (0.16)	− 0.21 (0.15)	− 0.19 (0.14)	− 0.21 (0.15)	− 0.22 (0.14)	− 0.20 (0.14)	− 0.22 (0.14)
Employment^e^	− 0.34 (0.15)*	− 0.28 (0.15)	− 0.40 (0.14)**	− 0.35 (0.14)*	− 0.24 (0.13)	− 0.30 (0.12)*	− 0.28 (0.12)*	− 0.24 (0.13)	− 0.33 (0.12)**	− 0.31 (0.12)*
Place of residence^f^										
Village	0.00 (0.21)	0.04 (0.21)	− 0.05 (0.21)	− 0.02 (0.21)	0.04 (0.17)	− 0.01 (0.17)	0.01 (0.17)	0.03 (0.17)	− 0.05 (0.17)	− 0.03 (0.17)
Town < 100,000 inhabitants	− 0.05 (0.17)	0.08 (0.16)	0.14 (0.16)	0.16 (0.16)	0.15 (0.15)	0.17 (0.15)	0.18 (0.15)	0.16 (0.15)	0.19 (0.15)	0.20 (0.15)
Psychiatric facility^g^	0.08 (0.18)	0.27 (0.17)	0.20 (0.17)	0.27 (0.17)	0.39 (0.16)*	0.35 (0.15)*	0.38 (0.16)*	0.40 (0.16)*	0.34 (0.15)*	0.37 (0.15)*
Duration of illness	− 0.10 (0.11)	− 0.07 (0.11)	− 0.10 (0.11)	− 0.09 (0.10)	− 0.07 (0.09)	− 0.08 (0.09)	− 0.07 (0.09)	− 0.07 (0.09)	− 0.09 (0.09)	− 0.08 (0.09)
Number of inpatient admissions	0.17 (0.10)	0.17 (0.10)	0.23 (0.11)*	0.22 (0.11)	0.24 (0.11)*	0.27 (0.11)*	0.26 (0.11)*	0.25 (0.10)*	0.25 (0.10)*	0.24 (0.10)*
Psychopathological symptoms (BPRS)	0.01 (0.08)	0.12 (0.08)	0.14 (0.07)	0.16 (0.08)*	0.15 (0.07)*	0.16 (0.06)*	0.17 (0.07)*	0.17 (0.07)*	0.17 (0.06)**	0.18 (0.06)**
**Depressive symptoms**										
Clinician-rated depression (CDSS)	–	− 0.34 (0.07)**	−	− 0.18 (0.07)*	− 0.15 (0.07)*	–	− 0.08 (0.07)	− 0.20 (0.07)**	–	− 0.09 (0.07)
Self-rated depression (CESD-R)	–	–	− 0.41 (0.07)**	− 0.32 (0.08)**	–	− 0.20 (0.07)**	− 0.16 (0.07)*	–	− 0.25 (0.07)**	− 0.74 (0.20)**
**Loneliness**										
DJGLS	–	–	–	–	− 0.52 (0.06)**	− 0.49 (0.06)**	− 0.48 (0.06)**	− 0.51 (0.06)**	− 0.46 (0.06)**	− 0.44 (0.06)**
**Interaction effects**										
DJGLS*CDSS	–	–	–	–	–	–	–	0.12 (0.05)*	–	–
DJGLS*CESD-R	–	–	–	–	–	–	–	–	0.15 (0.05)**	0.58 (0.20)**
*R* ^2^	0.22 (0.06)**	0.32 (0.06)**	0.31 (0.06)**	0.38 (0.06)**	0.53 (0.06)**	0.54 (0.05)**	0.54 (0.06)**	0.54 (0.06)**	0.56 (0.05)**	0.56 (0.06)**

Standardized coefficients are provided: STDYX for continuous variables and STDY for dichotomous variables. Standard errors (*SE*) are reported in parenthesis

*BPRS* Brief Psychiatric Rating Scale, *CDSS* Calgary Depression Scale for Schizophrenia, *CESD-R* Center for Epidemiologic Studies Depression Scale-Revised, *DJGLS* De Jong Gierveld Loneliness Scale

*P < 0.05, **P < 0.01

^a^0 = female, 1 = male; ^b^secondary = reference category; ^c^married/cohabiting = reference category; ^d^0 = living with someone, 1 = living alone; ^e^0 = employed/studying, 1 = unemployed; ^f^city > 100,000 inhabitants = reference category; ^g^0 = inpatient ward, 1 = outpatient clinic/day ward

Socio-demographic and clinical background variables (Model 1) jointly explained 22% of the variance in QoL. In the next two models, clinician-rated (CDSS) and self-rated (CESD-R) depression measures have been added separately. The effects of both CDSS (Model 2) and CESD-R (Model 3) on QoL were significant and negative, and an increase in explained variance was comparable (10 and 9%, respectively). When both depression measures were simultaneously entered to the model after socio-demographic and clinical factors (Model 4), the amount of variance accounted for rose more notably (by 16%) and the effect of CESD-R turned out to be higher than the effect of CDSS (although the latter was also significant). Subsequently, a loneliness measure (DJGLS) was added to the background variables and CDSS (Model 5), to the background variables and CESD-R (Model 6) and to the background variables and both CDSS and CESD-R (Model 7). This led to the reduction of the effects of both clinician- and self-rated depressive symptoms. In all cases, loneliness predicted worse QoL more strongly than depression measures and contributed substantially to the proportion of explained variation, which grew to 53% in Model 5 and to 54% in Models 6 and 7. After controlling for loneliness and self-rated depressive symptoms (Model 7), clinician-rated depression was no longer a significant predictor of QoL, whereas self-rated depression continued to predict lower QoL in all models.

**Fig. 2:**
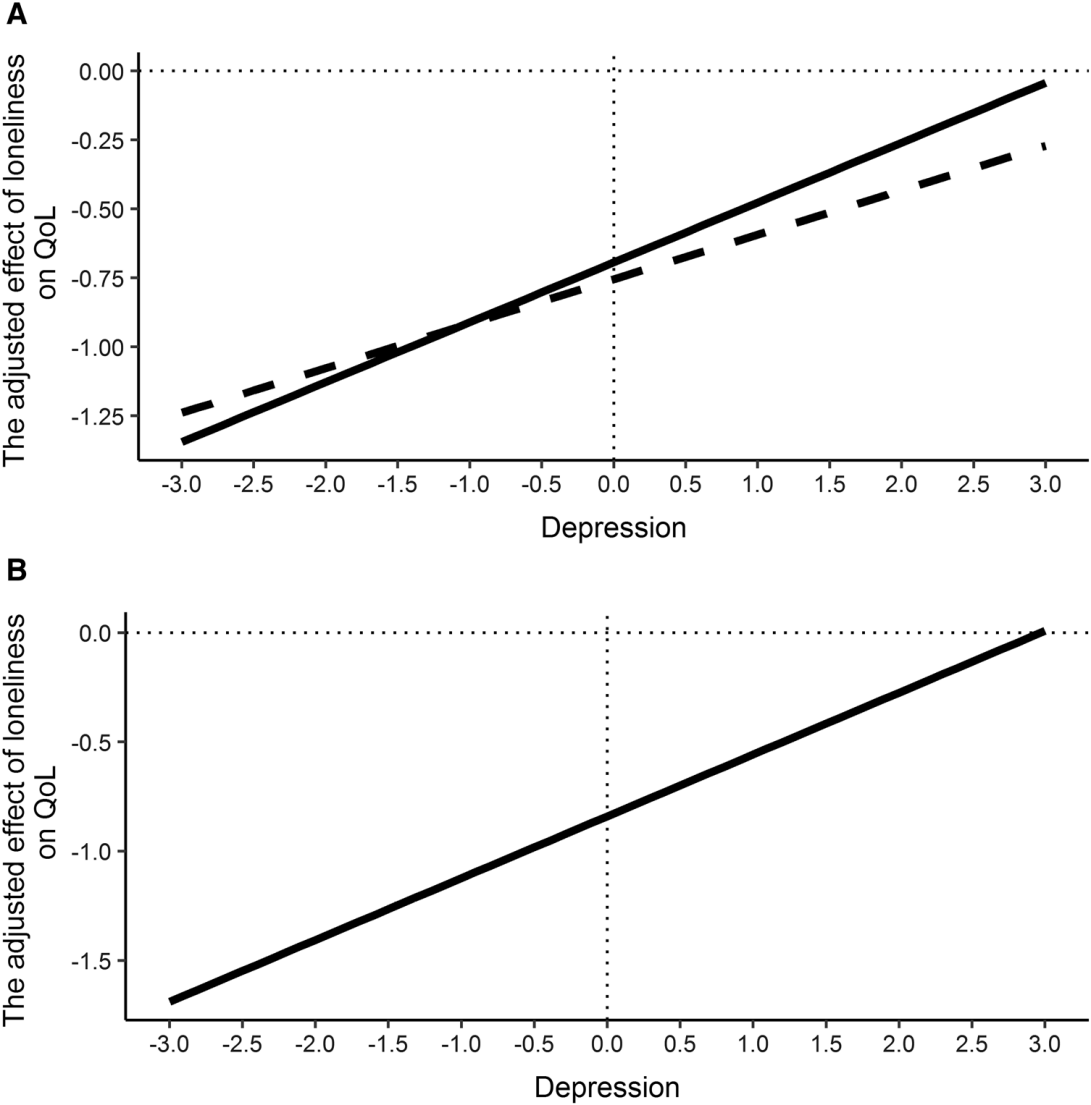
Interaction effects of measures of loneliness and depression on QoL. **A** Models 8 and 9: interaction effects of DJGLS and CDSS/CESD-R on SWLS; **B** Model 10: interaction effect of DJGLS and CESD-R on SWLS. Solid line represents the adjusted effect (the mean of the regression coefficient) of DJGLS on SWLS that corresponds to the full range of all continuous values of CESD-R. Dashed line represents the adjusted effect (the mean of the regression coefficient) of DJGLS on SWLS that corresponds to the full range of all continuous values of CDSS. *DJGLS* De Jong Gierveld Loneliness Scale,* CDSS* Calgary Depression Scale for Schizophrenia,* CESD-R* Center for Epidemiologic Studies Depression Scale-Revised,* SWLS* Satisfaction With Life Scale

Finally, in Models 8–10, the interaction effects of measures of loneliness and depression on QoL were introduced in the last step. These interactions are graphically represented in Fig. 2.

In Model 8 (including only a clinician-rated depression measure), the effect of the DJGLS on the SWLS was found to be moderated by the CDSS score (*β* = 0.12, *P* < 0.05). This result means that every time a person’s level of depression as measured by the CDSS rises by 1 SD, the adjusted effect of loneliness on QoL becomes more positive, increasing by 0.12 SD. Taking into account that loneliness negatively affects QoL (regression coefficient loneliness → QoL is negative), more positive values of loneliness mean de facto a reduction of the strength of its effect. In practice, this indicates that when the intensity of depressive symptoms is low, loneliness strongly impairs QoL; however, with a growing severity of depression the impact of loneliness on QoL becomes weaker.

Model 9 incorporated the CESD-R instead of the CDSS. Similar to the interaction of the DJGLS and the CDSS, the interaction of loneliness with self-rated depression also turned out to be statistically significant (*β* = 0.15, *P* < 0.01). As the level of depression grows, the effect of loneliness on QoL decreases (regression coefficient loneliness → QoL becomes more positive, i.e., approaches 0). In other words, analogically to the interaction DJGLS*CDSS, in the case of the interaction DJGLS*CESD-R the strength of the effect of loneliness on QoL also depends on the level of depression. It is greater when the severity of depressive symptoms is low and smaller when depressive symptoms are more pronounced.

In Model 10, both measures of depression have been included, however, since Model 7 revealed that after accounting for loneliness and self-rated depression, clinician-rated depression no longer significantly predicted QoL, only the interaction of the DJGLS with CESD-R was analyzed. Also, in this model, the interaction between loneliness and depression measured with the CESD-R proved to be significant (*β* = 0.58, *P* < 0.01). The positive value of the regression coefficient indicates that with an increasing severity of depressive symptoms the impact of loneliness on QoL becomes more positive. Taking into consideration the negative value of the regression coefficient between loneliness and QoL, this means that with the increase in the level of depression this coefficient gets closer to 0, so the impact of loneliness diminishes.

## Discussion

The data from this study provide evidence that higher levels of loneliness strongly predict worse subjective QoL in people with psychotic disorders, even after controlling for socio-demographics, clinical factors, and most importantly, depression. What is more, when not taking into account the interaction models, the negative effect of loneliness on QoL proved to be clearly larger than the effect of depression (both self-reported and clinician-rated), which is widely regarded as one of the core determinants of QoL in schizophrenia and related disorders [18–20]. This finding highlights the significance of satisfying social relationships for people with psychosis and supports the notion that loneliness may be a potent and devastating force in their lives, no less important than the clinical symptoms of the disease.

Interestingly, the markers of objective social isolation, which we included in the analyses as control variables (i.e., living circumstances and marital status) showed inconsistent associations with QoL. Namely, living alone was not significantly related to QoL, whereas being single, never married (as opposed to being married or cohabiting) robustly predicted lower QoL in each model. Taking these findings together, it is apparent that it is the type and quality of social relationships, and not the mere presence or absence of other people in an individual’s immediate environment that matters for his/her well-being.

As expected, depression also turned out to be an independent predictor of QoL. However, its negative effect on QoL was markedly reduced once loneliness was accounted for. This suggests that investigating the relationship of depressive symptoms with the QoL of persons with psychotic disorders without considering the role of the sense of loneliness may lead to an overestimation of their impact on QoL. Our results reveal as well that self-rated depression is more strongly associated with the subjective QoL in psychosis than clinician-rated depression. Furthermore, they show that the method of assessing depression may affect the general pattern of predictors of QoL (e.g., employment status was found to be associated with QoL in all models including self-rated depression, but not in models including only clinician-rated depression). It is also noteworthy that in a model not adjusted for loneliness (Model 4), after entering into the regression equation both measures of depression their main effects were independent of one another. Thus, each of these measures explained a slightly different aspect of QoL. This is additionally supported by the fact that the inclusion of the two measures simultaneously caused an increase in the proportion of the variation accounted for in the dependent variable in comparison to the previous models (i.e., 2 and 3), in which only one type of depression measure was incorporated. All this speaks in favor of the idea that clinician- and self-rated depression, although highly correlated and partially overlapping, are yet distinct constructs which may be somewhat differentially related to QoL in psychosis.

One possible explanation of the stronger association of the subjective QoL with patient-rated than with observer-rated depressive symptoms in our analyses may involve methodological issues related to the mode of administration of the measurement instruments. That is to say, the SWLS may be more highly related to the CESD-R than to the CDSS simply because the SWLS and the CESD-R are both self-report measures. However, other factors which may contribute to this difference should also be taken into consideration. For example, Hartman et al. [27] found that the presence of some forms of psychopathology in patients diagnosed with psychosis may lead to self-observer discrepancies in the assessment of depression. More specifically, patients with negative symptoms such as blunted affect or poor affective rapport tend to be rated as more depressed in clinician ratings compared with self-assessments. On the other hand, patients with more self-reported general psychopathology including anxiety or obsessive–compulsive symptoms are likely to over-report depression. It is, then, plausible that the association of various types of depression measures with subjective QoL in psychosis may be influenced to some extent by the overall symptom profile of the patients.

Very interesting and potentially clinically relevant findings were obtained from the analysis of the interaction effects of loneliness and depression on QoL. Namely, regardless of the type of depression measure used, depression moderated the relationship between loneliness and QoL in such a way that the negative effect of loneliness on QoL weakened with the increasing intensity of depressive symptoms. This seems to indicate that perceived deficits in social relationships are crucial for the subjective QoL in psychosis particularly among individuals with no or mild depression, whereas among those with more severe forms of depression other factors are likely to play a more prominent role. It should be noted, though, that since the main effect of loneliness remained significant after introducing the interaction effect, loneliness adversely affects QoL (although to a lesser degree) even when the level of depression is high.

The present study has implications for clinical practice. Although the results confirm the importance of incorporating interventions reducing depressive symptoms into therapeutic programs aiming to enhance QoL of people with psychotic disorders, at the same time they indicate that without addressing social factors such as loneliness the effectiveness of such programs may be limited. Furthermore, there is a need to tailor the content of the programs to the clinical status of patients. Given the moderating effect of depression on the relationship between loneliness and QoL, emphasis on alleviating loneliness should be placed first of all in the case of patients with low levels of depression, among whom the negative impact of loneliness on QoL is especially strong.

A recent review by Mann et al. [50] distinguished four broad groups of interventions directly targeting loneliness in people with mental health problems: changing cognitions, social skills training and psychoeducation, supported socialization or having a “socially-focused supporter”, and “wider community approaches”. The emerging evidence suggests that the most promising strategy is reducing maladaptive cognitions [50, 51]; however, in practice the selection of appropriate interventions should be made after careful consideration of such factors as the person’s residual disabilities and existing social network, the characteristics of the interventions, their effectiveness, the likelihood of a good fit between the person and the intervention, and the availability of interventions in the community [52].

Regarding research implications, the study findings show that the method chosen for measuring depression in models predicting QoL in psychosis matters. Future studies should further explore which aspects of each type of measurement account for the difference in the strength of the relationship with the subjective QoL between clinician- and self-assessed depression. It would be interesting to investigate whether the pattern of results would be different if both clinician- and self-rated depression measures were used as predictors of objective (i.e., observer-rated) QoL in people with psychotic disorders. This could help in responding to the question of the extent to which the stronger association of the subjective QoL with self-reported than with clinician-assessed depressive symptoms found in this study was due to common method variance.

The findings from this research must be interpreted in the light of several methodological limitations. First, the data are cross-sectional, which precludes any definite inferences about the direction of the relationships between variables. Second, it is possible that the fact that the patients were left on their own to fill in the self-report measures might have impacted the number of unanswered items. Next, the study participants were a convenience sample recruited from a single psychiatric institution, and therefore may not be representative of the entire population of people with psychotic disorders in Poland. The severity of depressive symptoms in this sample was rather low, which raises the possibility that their effect on QoL could have been underrated. Another limitation is that we did not utilize any formal measure of social networks to control for its effects. It should also be noted that self-assessment of QoL in psychosis is likely to be affected by factors not taken into account in this study, e.g., physical comorbidities, personality traits, cognitive functioning, insight, or medication used. Including them in the analyses may have influenced the strength of the relationships of loneliness and depression with QoL. Furthermore, we used an instrument assessing only a general satisfaction with life as a measure of subjective QoL, without considering satisfaction with specific life domains. Finally, although life satisfaction is one of the core components of QoL, its rating by people with severe mental illnesses may be potentially subject to a number of biases and is sometimes difficult to interpret [18, 53].

Despite these limitations, this study delivers useful insights into the harms caused by loneliness and depression to people with psychotic disorders. It lends support to the view that these two are related but distinct factors, demonstrates that they contribute strongly to the impairment of QoL of the patients and shows how they interact in this effect.
